# Atg7 in development and disease: panacea or Pandora’s Box?

**DOI:** 10.1007/s13238-015-0195-8

**Published:** 2015-09-24

**Authors:** Jianhua Xiong

**Affiliations:** Center for Molecular Medicine, National Heart, Lung and Blood Institute, National Institutes of Health, Bethesda, MD 20892 USA

**Keywords:** autophagy, Atg7, mouse model, development, disease

## Abstract

Macroautophagy is an evolutionarily conserved intracellular degradation system used by life ranging from yeasts to mammals. The core autophagic machinery is composed of ATG (autophagy-related) protein constituents. One particular member of the ATG protein family, Atg7, has been the focus of recent research. Atg7 acts as an E1-like activating enzyme facilitating both microtubule-associated protein light chain 3 (LC3)-phosphatidylethanolamine and ATG12 conjugation. Thus, Atg7 stands at the hub of these two ubiquitin-like systems involving LC3 and Atg12 in autophagic vesicle expansion. In this review, I focus on the pleiotropic function of Atg7 in development, maintenance of health, and alternations of such control in disease.

## **INTRODUCTION**

As a cellular scavenger, autophagy is a fundamental catabolic process consisting of three primary classes of autophagy: macroautophagy (the most prevalent form of autophagy and hereafter referred to as autophagy), microautophagy, and chaperone-mediated autophagy (Feng et al., 2015). Central to the sequential events of autophagy is *de novo* formation of cup-shaped isolation membranes (also known as phagophores) to sequester cytoplasmic components, expansion of this membrane to create a seal for a double membrane-bound vesicles called an autophagosome, and fusion of the autophagosome with a lysosome membrane to generate an autolysosome allowing degradation and recycling of the cargoes (Nakatogawa et al., 2009). Eukaryotic cells have evolved a well-organized autophagic machinery to adapt to and survive adverse microenvironmental conditions, including dwindling nutrient supplies (Galluzzi et al., 2014). Genetic screening of autophagy-deficient mutants in yeast provides us with almost 40 ATG (autophagy-related) genes, among which approximately 18 genes possess orthologues in higher eukaryotes. These ATG-encoded products act as the core autophagy machinery and contribute to the sequential steps of autophagosome formation including (I) induction of autophagosome formation by Atg1 complex, (II) phagophore expansion by Atg9-related cycling system, (III) vesicle nucleation by the phosphatidylinositol 3-kinase complex, and (IV) vesicle expansion by two ubiquitin-like conjugation systems. One such protein is Atg7, which is uniquely shared by, and plays crucial roles in, the two ubiquitin-like conjugation systems of microtubule-associated protein light chain 3 (LC3, a mammalian homologue of Atg8) and Atg12 respectively (Feng et al., 2015).

The ubiquitin-like conjugation system of LC3 involves Atg3, Atg4, Atg7, and LC3 for LC3-phosphatidylethanolamine production, while the ubiquitin-like conjugation system of Atg12 involves the Atg5, Atg7, Atg10, Atg12, and Atg16 for Atg12-Atg5-Atg16 production. The common ubiquitin E1-like activating enzyme, Atg7, is essential for the assembly and function of these two conjugates in the expansion of autophagosomal membranes (Nakatogawa et al., 2009; Feng et al., 2015). Substantial progress has been made during the past decade revealing the pivotal roles of Atg7 in autophagy-related cell homeostasis. Recent studies have unveiled the diverse and complex autophagy-dependent function of the evolutionarily conserved Atg7 in varying species, especially plants and animals. This review focuses on how this dynamic function is achieved and discusses the implications of altered Atg7-mediated autophagic activities in molecular, cellular, and organismal levels.

## **FUNCTION OF ATG7 IN PLANTS**

Phenotypic analyses of Atg7 mutants indicates that Atg7 disruption renders Arabidopsis (*Arabidopsis thaliana*) cells hypersensitive to a shortage of nutrients with features of premature leaf senescence, though the mutant is otherwise normal (Doelling et al., 2002). Increased expression of multiple LC3 isoforms are observed in Atg7 mutants due to impaired control of the two ubiquitin-like conjugation systems of LC3 and Atg12 (Thompson et al., 2005). Deletion of Atg7 in *Nicotiana benthamiana* and Arabidopsis leads to unrestricted hypersensitive responses during plant innate immunity (Liu et al., 2005; Hofius et al., 2009). Arabidopsis Atg7 mutant is also more susceptible to fungal infection (Lenz et al., 2011). Minina and colleagues showed that the autotroph Arabidopsis can benefit from caloric restriction-induced lifespan extension via Atg7-regulated autophagy (Minina et al., 2013).

## **FUNCTION OF ATG7 IN INVERTEBRATES**

### **Nematode**

A similar role of Atg7-regulated autophagy in dietary restriction-induced lifespan extension exists in *Caenorhabditis elegans* (*C. elegans*) (Jia and Levine, 2007). In addition, genetic inactivation of *C. elegans* Atg7 exacerbates accumulation of toxic polyglutamine expansion protein aggregates and accelerates progress of neurodegenerative disorders (Jia et al., 2007).

### **Fruit fly**

Steroid- and radiation-triggered programmed cell death accompanies increased Atg7 transcripts in Drosophila cells (Lee et al., 2003). Normal levels of Atg7-modulated autophagy, albeit dispensable for metamorphosis, seem to be critical for preventing neurodegeneration, resisting stresses, and promoting longevity in Drosophila (Juhasz et al., 2007; Juhasz and Neufeld, 2008). Using the Drosophila eye as a model system, Chen et al. described Atg7 as a downstream effector of heat shock protein 27, and as a participant in the regulation of normal eye development, neuronal homeostasis, and lifespan (Chen et al., 2012). Such critical roles of fruit fly Atg7 has been reported not only in development but also in infection. *Mycobacterium marinum* infection is sufficient to counteract the effectiveness of antimycobacterial treatment, and thereby drastically affects the survival rate in Atg7 mutant Drosophila (Kim et al., 2012). In addition, a recent study reveals a novel example of Atg7-independent autophagy during the developmental shortening of Drosophila intestine (Chang et al., 2013).

## **FUNCTION OF ATG7 IN ZEBRAFISH AND RATS**

In zebrafish, approximately one third of Atg7-knockdown morphants had ectopic expression of essential transcription factors and severe developmental defects in cardiac morphology encompassing heart looping, pericardial edema, and malformation of chamber and valve (Lee et al., 2014). Gain- and loss-of-function of Atg7 studies in the αB-crystallin R120G mutation (CryAB^R120G^) model of rat desmin-related cardiomyopathy reveal the significant ability of ATG7 in reversing autophagic deficiency and maintaining physiological levels of basal autophagy (Pattison et al., 2011). As a consequence of Cathepsin B treatment, stimulated ATG7-mediated autophagy aggravates lipotoxicity via induction of nod-like receptor 3 proinflammatory response in rat insulinoma cells (Li et al., 2013).

## **FUNCTION OF ATG7 IN MICE**

To investigate the *in vivo* function of ATG7 in mammals, Komatsu et al. generated Atg7-deficient mice (Atg7^−/−^). As anticipated, Atg7^−/−^ mice exhibit impaired constitutive and starvation-induced autophagy; however, they die soon after birth (Komatsu et al., 2005). Therefore, Ubc-CreERT2 mice were crossed with Atg7-floxed (Atg7^flox/flox^) mice for the generation of tamoxifen-inducible whole body Atg7 knockout mice. Karsli-Uzunbas et al. further reported that acute systemic deletion of Atg7 in adult mice leads to perturbed glucose metabolism, but blocks the progression of non-small cell lung cancer (NSCLC) *in vivo* (Karsli-Uzunbas et al., 2014). Thus, cells from embryo/fetus of Atg7^−/−^ mice and certain cell/tissue-specific Atg7-knockout postnatal mice were extensively employed in the quest for understanding the mechanisms underlying the pleiotropic effects of ATG7 in development, physiology, and pathology (Table 1).

**Table 1:** **Function of Atg7 revealed by genetic mouse models.**

Atg7-knockout targets	Tools	Predominant phenotypes	References
Whole-body	Zp3-Cre	Postnatal lethality; impaired bacteria clearance, unaffected obatoclax-induced toxicity, augmented genomic instability, aberrant cell apoptosis, and altered cell cycle, and cytoskeletal protein filamentous actin network in mouse embryonic fibroblasts	Komatsu et al., 2005; Sun et al., 2008; McCoy et al., 2010; Lee et al., 2012; Zhuo et al., 2013
	iUbc-Cre	Perturbed glucose metabolism and inhibited progression of non-small cell lung cancer	Karsli-Uzunbas et al., 2014
Liver	Mx1-Cre	Hepatomegaly with malformations of organelles and ubiquitin-positive protein aggregates	Komatsu et al., 2005; Matsumoto et al., 2008
	Alb-Cre	Oxidative stress with increased total protein mass; excessive storage of triglyceride in lipid droplets during nutrient deprivation	Matsumoto et al., 2008; Singh et al., 2009a
	GFAP-Cre	Inhibited lipid release and fibrogenesis in hepatic stellate cells	Hernandez-Gea et al., 2012
Pancreas	RIP-Cre	Impaired glucose tolerance; degenerated islets; decreased mitochondrial oxidation consumption and increased compensatory basal glycolytic rates and reactive oxygen species levels	Ebato et al., 2008; Jung et al., 2008; Wu et al., 2009
Skeletal muscle	MCK-Cre	Decreased mitochondrial oxidation consumption and increased compensatory basal glycolytic rates and reactive oxygen species levels	Wu et al., 2009
Endothelium	VE-cadherin-Cre	Impaired von Willebrand factor (VWF) release; susceptibility to bleomycin-induced pulmonary fibrosis	Torisu et al., 2013; Singh et al., 2015
Vascular smooth muscle	SM22α-Cre	Sarcoplasmic reticulum swelling and imbalanced Ca^2+^ homeostasis	Michiels et al., 2015
Adipose	Fab4 (aP2)-Cre	Lean body mass and acquisition of brown adipose tissue features	Zhang et al., 2009a; Singh et al., 2009b
Mammary gland	WAP-Cre	Impaired keratin 8 homeostasis; defective phagocytosis and enhanced inflammatory responses	Kongara et al., 2010; Teplova et al., 2013
Neuron	Nestin-Cre	Neurodegenerative symptoms	Komatsu et al., 2006a
	Pcp2-Cre	Axonal dystrophy	Komatsu et al., 2007
	POMC-Cre	Elevated lipolysis; dysregulation of metabolic modulation	Kaushik et al., 2012; Coupe et al., 2012
	CamKII-Cre	Neurodegenerative symptoms	Inoue et al., 2012a; Nilsson et al., 2013
	Cre-expressing viruses	Aberrant inflammation responses; repressed retrograde degeneration of dopaminergic axons	Cheng et al., 2011; Motori et al., 2013
	VAChT-Cre	No apparent phenotypes of amyotrophic lateral sclerosis	Tashiro et al., 2012
	DAT-Cre	Neurodegenerative symptoms; altered dopaminergic axonal profile and morphology	Inoue et al., 2012a; Hernandez et al., 2012
Bone marrow/Hematopoiesis	EIIa-Cre	Impaired mitochondrial clearance during reticulocyte maturation	Zhang et al., 2009b
	Lck-Cre	Aberrant production of IL-2 and IFN-γ; impaired stimulated proliferation, endoplasmic reticulum homeostasis, and calcium mobilization	Hubbard et al., 2010; Jia et al., 2011
	Vav-iCre	Severe and fatal anemia and myeloproliferation; impaired response to α-herpesviruses infection and viral DNA recognition; compromised macrophagic differentiation induction and function acquisition	Mortensen et al., 2010, 2011; Rasmussen et al., 2011; Jacquel et al., 2012
Intestine	Villi-Cre or Villi-CreER	Elevated inflammatory responses; promoting tumorigenesis; impaired immune homeostasis; damaged Paneth cells	Cadwell et al., 2009; Fujishima et al., 2011; Wittkopf et al., 2012; Inoue et al., 2012b; Nishiumi et al., 2012; Adolph et al., 2013
Skin	K14-Cre	Impaired removal of reactive oxidized phospholipids and damaged protein aggregates; dispensible for skin barrier function	Zhao et al., 2013; Rossiter et al., 2013
Kidney	PEPCK-Cre	Vulnerable to cisplatin- and ischemia-reperfusion induced acute renal injury	Jiang et al., 2012

### **Embryonic fibroblasts**

Wild-type (WT) mouse embryonic fibroblasts (MEFs) were used to recapitulate robust autophagy-mediated capability of bacteria clearance, which is absent in Atg7^−/−^ MEFs (Sun et al., 2008). Using WT and Atg7^−/−^ MEFs and small interference RNA (siRNA)-mediated silencing of Atg7 in BAX/BAK-knockout MEFs, it has been demonstrated that Atg7-regulated autophagy is dispensable for obatoclax-induced toxicity (McCoy et al., 2010). Subsequently, Lee et al. found a novel function of Atg7, independent of its E1-like enzymatic activity. Briefly, ATG7 coordinates tumor suppressor p53-mediated cell division cycle and cell apoptosis via physical interaction with p53 under limited nutrients, providing an explanation for the simultaneous or sequential metabolic stress-induced events, including exit from cell cycle, induction of autophagy, and activation of cell death signaling. In addition, the augmented genomic instability in Atg7^−/−^ mice may be a reason for its postnatal death (Lee et al., 2012). To characterize the regulatory network of autophagy, quantitative iTRAQ labeling coupled with on-line 2D LC/MS/MS proteomics analysis was performed in WT and Atg7^−/−^ MEFs. The result implied that basal and starvation-induced autophagy depends on an intact cytoskeletal protein filamentous actin network (Zhuo et al., 2013).

### **Liver cells**

Mx1-Cre transgenic mice were crossed with Atg7-floxed (Atg7^flox/flox^) mice for the generation of hepatocyte-specific polyinosinic acid-polycytidylic acid-inducible Atg7 knockout (iMx1-Atg7^−/−^) mice (Komatsu et al., 2005; Matsumoto et al., 2008). iMx1-Atg7^−/−^ mice present hepatomegaly with malformations of organelles and ubiquitin-positive protein aggregates (Komatsu et al., 2005). Alb-Cre mice were crossed with Atg7^flox/flox^ mice for the generation of hepatocyte-specific Atg7 knockout (Alb-Atg7^−/−^) mice (Matsumoto et al., 2008; Singh et al., 2009a). Comprehensive proteomics analyses of iMx1-Atg7^−/−^ and Alb-Atg7^−/−^ mice and their controls suggest that autophagy-deficient hepatic cells exert oxidative stress with increased total protein mass, specifically glutathione S-transferase families, protein disulfide isomerase, and glucose-regulated proteins (Matsumoto et al., 2008). Alb-Atg7^−/−^ mice also showed higher triglyceride storage in lipid droplets during nutrient deprivation than controls, providing evidence that lipolysis and autophagy are interrelated through macrolipophagy (Singh et al., 2009a). GFAP (glial fibrillary acid protein)-Cre mice were crossed with Atg7^flox/flox^ mice for the generation of hepatic stellate cell-specific Atg7 knockout (GFAP-Atg7^−/−^) mice. A surprising detrimental consequence of autophagy in deteriorating hepatic fibrogenesis through release of lipids from activated stellate cells, has been established in GFAP-Atg7^−/−^ mice *in vivo* and the mouse immortalized stellate cell line JS1 *in vitro* (Hernandez-Gea et al., 2012).

### **Pancreatic β cells and skeletal muscle cells**

RIP-Cre mice were crossed with Atg7^flox/flox^ mice for the generation of pancreatic β cell-specific Atg7 knockout (β cell-Atg7^−/−^) mice (Ebato et al., 2008; Jung et al., 2008; Wu et al., 2009). These mice display impaired glucose tolerance and degenerated islets accompanied by reduced β cell mass and insulin secretion levels. A series of morphological malformations occur in Atg7 mutant β cells, including accumulation of ubiquitinated inclusions, enlargement of mitochondria, and distension of the endoplasmic reticulum (Ebato et al., 2008; Jung et al., 2008). MCK-Cre mice were crossed with Atg7^flox/flox^ mice for the generation of skeletal muscle cell-specific Atg7 knockout (SMC-Atg7^−/−^) mice. Furthermore, Wu et al. observed a decrease of mitochondrial oxidation consumption and an increase of compensatory basal glycolytic rates and reactive oxygen species levels in cells derived from β cell-Atg7^−/−^ and SMC-Atg7^−/−^ mice (Wu et al., 2009).

### **Endothelial cells and vascular smooth muscle cells**

VE-cadherin-Cre transgenic mice were crossed with Atg7^flox/flox^ mice for the generation of endothelial cell-specific Atg7 knockout (EC-Atg7^−/−^) mice (Torisu et al., 2013; Singh et al., 2015). Compared to WT littermate controls, EC-Atg7^−/−^ mice have impaired von Willebrand factor (VWF) release elicited by epinephrine, implying a promising strategy for transient prevention of thrombosis (Torisu et al., 2013). Moreover, Atg7-null endothelial cells also confer susceptibility to bleomycin-induced pulmonary fibrosis *in vivo* by endothelial-to-mesenchymal transition (EndMT) (Singh et al., 2015).

SM22α-Cre transgenic mice were crossed with Atg7^flox/flox^ mice for the generation of vascular smooth muscle cell-specific Atg7 knockout mice. Vascular smooth muscle cell-specific Atg7 deletion leads to sarcoplasmic reticulum swelling and imbalanced Ca^2+^ homeostasis, resulting in altered contractility (Michiels et al., 2015).

### **Fat cells and mammary gland cells**

Fab4 (aP2)-Cre mice were crossed with Atg7^flox/flox^ mice for the generation of adipocyte-specific Atg7 knockout (FC-Atg7^−/−^) mice. Targeted deletion of Atg7 in adipose tissues leads to a lean body mass with an elevated rate of β-oxidation and a low rate of lipolysis. The white adipose tissue in FC-Atg7^−/−^ mice acquired more features of brown adipose tissue, and its mass diminished (Zhang et al., 2009a; Singh et al., 2009b). Strikingly, disruption of ATG7 confers sensitivity to insulin stimuli (Zhang et al., 2009a). Additional evidence that ATG7 plays a vital role in adipogenesis has been obtained in 3T3-L1 preadipocytes, wherein inhibition of ATG7 hampered adipocyte differentiation and lipid accumulation (Singh et al., 2009b).

WAP-Cre mice were crossed with Atg7^flox/flox^ mice for the generation of mammary gland cell-specific Atg7 knockout (MGC-Atg7^−/−^) mice (Kongara et al., 2010; Teplova et al., 2013). Using MGC-Atg7^−/−^ mice, Kongara et al. linked ATG7-regulated autophagy to limiting ER and oxidative stress and orchestrating keratin 8 homeostasis in mammary cells (Kongara et al., 2010). Besides this phenotype, MGC-Atg7^−/−^ mice undergo defective phagocytosis, compromised dead cell clearance, and enhanced inflammatory responses in mammary involution, reminiscent of tumor-modulating niche and ductal ectasia. Consistent with these observations, specific knockdown of Atg7 in immortalized mouse mammary epithelial cells strengthened the conclusion that ATG7 is needed for effective dead cell engulfment (Teplova et al., 2013).

### **Neurons**

(I) Nestin-Cre transgenic mice were crossed with Atg7^flox/flox^ mice for the generation of neuron-specific Atg7 knockout (nestin-Atg7^−/−^) mice (Komatsu et al., 2006a). Consistent with the findings from invertebrates (Jia et al., 2007; Juhasz et al., 2007; Juhasz and Neufeld, 2008; Chen et al., 2012), nestin-Atg7^−/−^ mice lacking autophagy in the central nervous system displayed a broad range of neurodegenerative symptoms, including accumulation of inclusion bodies in Atg7-deletion neurons, loss of massive neurons in the cerebral and cerebellar cortices, and defects of behavioral coordination (Komatsu et al., 2006a). (II) Pcp2-Cre transgenic mice were crossed with Atg7^flox/flox^ mice for the generation of Purkinje cell-specific Atg7 knockout mice. Similar to nestin-Atg7^−/−^ mice, Purkinje cell-specific loss of Atg7 function impeded autophagy-related membrane trafficking and turnover resulting in axonal dystrophy, a sign of axonopathy associated with neurodegenerative disease (Komatsu et al., 2007). (III) POMC (pro-opiomelanocortin)-Cre transgenic mice were crossed with Atg7^flox/flox^ mice for the generation of POMC neuron-specific Atg7 knockout (POMC-Atg7^−/−^) mice (Kaushik et al., 2012; Coupe et al., 2012). In POMC-Atg7^−/−^ mice, Kaushik et al. drew a consistent conclusion by a previous study in Alb-Atg7^−/−^ mice that autophagy negatively regulates lipolysis (Singh et al., 2009a; Kaushik et al., 2012). Moreover, direct genetic evidence was obtained that ATG7 participates in normal development and metabolic modulation in POMC neurons, indicating potential roles of Atg7 deficiency in the pathogenesis of obesity and aging-related metabolic syndrome (Kaushik et al., 2012; Coupe et al., 2012). (IV) CamKII-Cre transgenic mice were crossed with Atg7^flox/flox^ mice for the generation of forebrain neuron-specific Atg7 knockout (CamKII-Atg7^−/−^) mice. Remarkably, protective roles of Atg7 in neurodegeneration of forebrain neurons have been elucidated in CamKII-Atg7^−/−^ mice (Inoue et al., 2012a; Nilsson et al., 2013). Atg7 ablation correlates with the progression of age-dependent neurodegeneration via tau phosphorylation pathway (Inoue et al., 2012a). By breeding CamKII-Atg7^−/−^ mice with amyloid precursor protein transgenic mice, Nilsson et al. found that autophagy deficiency led to reduced amyloid beta (Aβ) secretion and concurrent accumulation of intracellular Aβ peptide, indicative of Alzheimer’s disease (Nilsson et al., 2013). (V) Atg7 knockout astrocytes failed to orchestrate intricate mitochondria network for normal inflammation responses (Motori et al., 2013). In addition, VAChT-Cre mice were crossed with Atg7^flox/flox^ mice for the generation of motor neuron-specific Atg7 knockout (VAChT-Atg7^−/−^) mice. Using VAChT-Atg7^−/−^ mice, Tashiro et al. exclude the potential involvement of autophagy in the pathogenesis of amyotrophic lateral sclerosis (Tashiro et al., 2012). In contrast, ATG7-mediated autophagy as an upstream cell death driver controls lysosomal dysfunction-induced cell apoptosis in mouse C17.2 neural stem cells (Walls et al., 2010).

Interestingly, seemingly opposing effects of ATG7 in dopamine neurons have been delineated by different groups (Cheng et al., 2011; Inoue et al., 2012a; Hernandez et al., 2012). DAT-Cre mice were crossed with Atg7^flox/flox^ mice for the generation of dopamine neuron (enriched in the substantia nigra pars compacta)-specific Atg7 knockout (DAT-Atg7^−/−^) mice (Inoue et al., 2012a; Hernandez et al., 2012). Inoue et al. observed that DAT-Atg7^−/−^ exhibits an even more severe phenotype of age-dependent neurodegeneration than CamKII-Atg7^−/−^ mice (Inoue et al., 2012a). Conversely, conditional deletion of Atg7 in substantia nigra dopaminergic neurons of adult mice by intranigral injection of adeno-associated virus-Cre achieves unexpected protection in retrograde degeneration of dopaminergic axons. Additionally, this process is tightly controlled by Akt/Rheb/the kinase mammalian target of rapamycin (mTOR) signaling pathways (Cheng et al., 2011). Likewise, using DAT-Atg7^−/−^ mouse model, Hernandez et al. revealed that mTOR inhibitor rapamycin decreases evoked dopamine secretion and decelerates recovery in Dopamine neurons, which is an autophagy-dependent regulation of presynaptic neurotransmission (Hernandez et al., 2012).

### **Hematopoietic cells**

(I) EIIa-Cre mice were bred with Atg7^flox/flox^ mice, and the progeny heterozygous Atg7^+/−^ mice were intercrossed for the generation of E13.5 homozygous Atg7^−/−^ mice. Then, transplantation of E13.5 Atg7^−/−^ fetal liver cells into H2K-GFP mice was carried out to examine the hematopoietic lineages. A novel finding was observed that Atg7-dependent and independent mechanisms contribute to mitochondrial clearance during reticulocyte maturation (Zhang et al., 2009b). (II) Lck-Cre mice were crossed with Atg7^flox/flox^ mice for the generation of T cell-specific Atg7 knockout (Lck-Atg7^−/−^) mice (Hubbard et al., 2010; Jia et al., 2011). Based on analyses of Lck-Atg7^−/−^ mice, it has been reported that that autophagy is responsible for maintenance of normal production of IL-2 and IFN-γ, stimulated proliferation, endoplasmic reticulum homeostasis, and calcium mobilization, in T lymphocytes; nevertheless, T cells derived from Lck-Atg7^−/−^ mice had no detectable increased apoptosis (Hubbard et al., 2010; Jia et al., 2011). The very slow activation-induced proliferation makes it difficult to differentiate polarized Th1 cell populations. To this end, Cre-ER mice were crossed with Atg7^flox/flox^ mice for the generation of tamoxifen-inducible Atg7 knockout (ER-Atg7^−/−^) mice. Unsurprisingly, deletion of Atg7 in isolated T cells from ER-Atg7^−/−^ mice resulted in decreased activation-induced cytokine production (Hubbard et al., 2010). (III) Vav-iCre mice were crossed with Atg7^flox/flox^ mice for the generation of hematopoietic system-specific Atg7 knockout (Vav-Atg7^−/−^) mice (Mortensen et al., 2010, 2011; Rasmussen et al., 2011; Jacquel et al., 2012). Loss of Atg7-mediated autophagy hampered mitochondria removal and erythroid development, as well as proliferation and genomic integrity of hematopoietic stem cells, giving rise to severe and fatal anemia and myeloproliferation in Vav-Atg7^−/−^ mice (Mortensen et al., 2010; 2011). In addition to these functions, bone marrow-derived dendritic cells from Vav-Atg7^−/−^ mice mitigated the response to α-herpesvirus infection and viral DNA recognition due to reduction of ATG7-dependent IFN-β expression (Rasmussen et al., 2011). *Ex vivo* assessment of monocytes from Vav-Atg7^−/−^ mice indicates that macrophagic differentiation induction and function acquisition could be attributed to ATG7-mediated autophagy (Jacquel et al., 2012).

### **Intestinal cells**

Villi-Cre (or Villi-CreER) transgenic mice were crossed with Atg7^flox/flox^ mice for the generation of intestinal epithelium-specific (tamoxifen-inducible) Atg7 knockout (Villi-Atg7^−/−^) mice (Cadwell et al., 2009; Fujishima et al., 2011; Wittkopf et al., 2012; Inoue et al., 2012b; Nishiumi et al., 2012; Adolph et al., 2013). Like its orthologues Atg16L1 and Atg5, Atg7 aids in the normal morphology, and granule formation and exocytosis of Paneth cells, as suggested by analyses of this mouse model (Cadwell et al., 2009; Wittkopf et al., 2012). Villi-Atg7^−/−^ mice displayed upregulated gene expression associated with inflammation and, thereby, endotoxin or *Citrobacter rodentium*-induced inflammatory responses via NF-κB inactivation (Cadwell et al., 2009; Fujishima et al., 2011; Inoue et al., 2012b). These observations are underscored by another seminal mouse genetic work showing that Villi-Atg7^−/−^ mice synergistically with intestinal epithelium-specific Xbp1-deficient mice recapitulates features of Crohn’s disease, as a specific type of Paneth cell disease (Adolph et al., 2013). Nonetheless, thus far, no overt evidence has been obtained from Villi-Atg7^−/−^ mice that Atg7 is implicated in the pathogenesis of intestinal tumors and maintenance of gut immune homeostasis (Nishiumi et al., 2012; Wittkopf et al., 2012).

### **Skin cells, kidney cells, and cardiomyocytes**

K14-Cre mice were crossed with Atg7^flox/flox^ mice for the generation of epidermal keratinocyte-specific Atg7 knockout mice (Zhao et al., 2013; Rossiter et al., 2013). Using this mouse model, Zhao et al. highlighted the importance of ATG7 for the removal of reactive oxidized phospholipids and damaged protein aggregates in the epidermis exposed to environmental insults (Zhao et al., 2013). However, ATG7-mediated autophagy appears to be nonessential to execute skin barrier function (Rossiter et al., 2013). ATG7 has also been documented as a core regulator in caspase-8 inhibition-induced autophagic cell death in mouse L929 skin fibroblast cells (Yu et al., 2004).

PEPCK-Cre mice were crossed with Atg7^flox/flox^ mice for the generation of kidney proximal tubular cell-specific Atg7 knockout (PEPCK-Atg7^−/−^) mice. These autophagy-deficient PEPCK-Atg7^−/−^ mice are particularly vulnerable to cisplatin- and ischemia-reperfusion induced acute renal injury, suggesting potent renal protection by ATG7-mediated autophagy (Jiang et al., 2012).

To test whether autophagy can ameliorate or restore proteinopathy in CryAB^R120G^ cardiac model of cardiomyopathy, Bhuiyan et al. crossed ATG7-expressing mice and CryAB^R120G^ mice to generate Atg7-crossed CryAB^R120G^ mice. Indeed, the entire cohort of Atg7-crossed CryAB^R120G^ mice acquire relatively sustained autophagy, leading to improved cardiac function (Bhuiyan et al., 2013).

## **FUNCTION OF ATG7 IN HUMAN**

**Figure 1:**
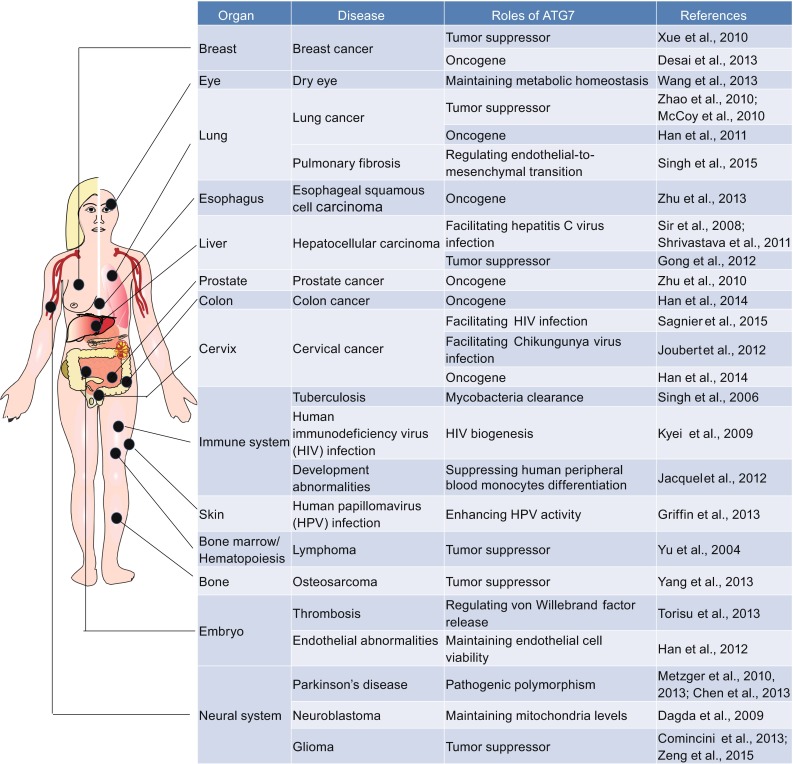
**Schematic illustration of physiopathological roles of ATG7 in human**

Prompted by the clues from model organisms, the architecture of the functional ATG7-mediated regulatory network has been explored in the settings of human biology and disease, such as cancer, infectious disease, and neurodegenerative diseases (Fig. 1).

### **Cancer**

As implied earlier, autophagy has been considered as both a pro-survival pathway and type 2 cell death (Kroemer and Levine, 2008). Two main hallmarks of cancer cells are unrestricted proliferation and suppressed cell death (Hanahan and Weinberg, 2011), raising the possibility of ATG7 as both an oncogene and a tumor suppressor. On one hand, ATG7 suppresses resistance of human breast cancer cells to photodynamic therapy (Xue et al., 2010). It also facilitates the anti-tumor actions of cytosolic FoxO1 and obatoclax in human NSCLC cells (Zhao et al., 2010; McCoy et al., 2010), compound 2-Methoxyestradiol in human osteosarcoma (Yang et al., 2013), and tetrandrine in human hepatocellular carcinoma (Gong et al., 2012). Moreover, the caspase-8 inhibition-initiated autophagic cell death program requires ATG7 via activation of receptor-interacting protein/c-Jun N-terminal kinase signaling in human U937 monocyte lymphoma cells (Yu et al., 2004). Additionally, two microRNAs, miR-17 and miR-137, have been shown to target ATG7 for acquisition of resistance to anticancer drugs and low-dose ionizing radiation treatments in human glioma cells (Comincini et al., 2013; Zeng et al., 2015). On the other hand, ATG7 can also serve as an oncogene. Heat shock factor 1-controlled transcriptional expression of ATG7 is inversely correlated with the chemotherapeutic prognosis of breast cancer patients (Desai et al., 2013). Inhibition of redundant Atg7-mediated lysosome-autophagy pathway augments the anti-cancer effects of a proteasome inhibitor in some human prostate cancer cells (Zhu et al., 2010), epidermal growth factor receptor-tyrosine kinase inhibitors in human lung cancer cells (Han et al., 2011), and cisplatin in human esophageal squamous cell carcinoma cells (Zhu et al., 2013). Through reciprocal mechanical interaction, ATG7, rather than ATG5 and Beclin-1, represses caspase-9-mediated apoptosis in human colon and cervical cancer cells. Caspase-9 promotes Atg7-mediated autophagy (Han et al., 2014). Notably, ATG7 has a relatively higher expression in human THP1 acute monocytic leukemia cells than a panel of human immune and epithelial cells (Rioux et al., 2007). Importantly, two physical interactions between ATG7 and acetyltransferase p300 and between ATG7 and transcription factor p53 have been depicted in human HeLa cervical and HCT116 colon cancer cells, respectively, in the context of limited nutrient availability (Lee and Finkel, 2009; Lee et al., 2012).

### **Infectious disease**

Dual effects of ATG7-mediated autophagy intersection with human immunodeficiency virus (HIV) biogenesis fuel the viral yields as they do in human U937 monocytoid cells and in primary human macrophages (Kyei et al., 2009). A battery of morphological and biochemical assays have been conducted showing that hepatitis C virus (HCV) causes an unfolded protein response-dependent incomplete ATG7-mediated autophagy during pathogenesis in human hepatoma cells (Sir et al., 2008). Also, it has been documented that disruption of ATG7-mediated autophagy can evoke the interferon signaling pathway resulting in apoptosis of HCV-infected immortalized human hepatocytes (Shrivastava et al., 2011), and dramatically enhanced infectivity of human papillomavirus in primary human keratinocytes (Griffin et al., 2013). Knockdown of Atg7 also interferes with the elimination of intracellular pathogen *Mycobacterium tuberculosis* by human immunity-related GTPase family M protein in U937 cells (Singh et al., 2006). ATG7-mediated autophagy is also involved in the constricting activity of HIV infection via release of the HIV-1 transactivator Tat in human embryonic kidney 293 cells and MAGIC5B cells (i.e., HeLa cells modified to express CD4 and CXCR4 together with β-galactosidase under the control of the HIV LTR promoter) (Sagnier et al., 2015). Silencing of ATG7 may delay the progression of Chikungunya virus-induced caspase-dependent cell death in human fibroblast cells and HeLa cells (Joubert et al., 2012), and suppress the colony stimulating factor-1-induced differentiation of human peripheral blood monocytes into macrophages (Jacquel et al., 2012).

### **Neurodegenerative disease**

It has been shown that downregulation of ATG7 can compensate the loss of mitochondria in PTEN-induced kinase 1 (PINK1) deficient dopaminergic human neuroblastoma cells, likely supporting the potential role of ATG7 in PINK1 mutation-related familial Parkinson’s disease (Dagda et al., 2009). By analyzing a large number of European Huntington disease patients, Metzger et al. found that the V471A polymorphism in ATG7 was significantly associated with the age at onset. More specifically, the V471A polymorphism in ATG7 correlates with an earlier disease onset of 4 years in a mixed group of Huntington disease populations (Metzger et al., 2010, 2013). In five patients with Parkinson’s disease, four novel genetic variants including 11313449G>A, 11313811T>C, 11313913G>A, and 11314041G>A, were identified on the ATG7 gene promoter, implying the altered transcriptional activity of the ATG7 may be a risk factor (Chen et al., 2013).

### **Miscellaneous**

Together with FOXO3-ATG101 complex, coupling of acetylated FOXO1 with ATG7, upon stimulation with prosecretory mitogen lacritin, can rescue the metabolic homeostasis in human corneal epithelial cells (Wang et al., 2013). In a similar manner, acetylated FOXO1 and ATG7 can preserve human umbilical vein endothelial cells (HUVECs) viability under circumstances of oxidative stress (Han et al., 2012). ATG7 is also essential for normal secretion of VWF in HUVECs (Torisu et al., 2013), and EndMT in both HUVECs and human pulmonary aortic endothelial cells (Singh et al., 2015).

## **CONCLUDING REMARKS**

**Figure 2:**
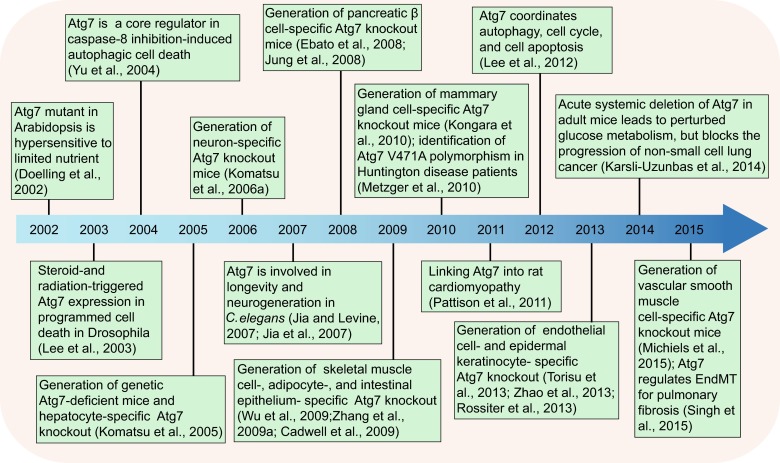
**Retrospective analyses of major events of Atg7 research in development and disease**

The word “autophagy”, literally auto-, meaning “self”, and phagein, meaning “to eat”, in Greek, was originally coined by Belgian cytologist Christian de Duve in 1963 (Klionsky, 2008). More than 50 years have passed since autophagy was defined as a core mechanism underlying both elimination and recycling of intracellular materials in normal development and diverse disease categories (Mizushima and Komatsu, 2011; Choi et al., 2013; Murrow and Debnath, 2013). These include, but are not limited to, immunity (Virgin and Levine, 2009), metabolism (Codogno and Meijer, 2010; Rabinowitz and White, 2010), aging (Madeo et al., 2010), and the cardiovascular (De Meyer et al., 2015; Nussenzweig et al., 2015), and nervous system (Komatsu et al., 2006b). Although still in the early stages, it appears to be almost clear how core machinery plays in Atg7-dependent and -independent autophagosome biogenesis (Nishida et al., 2009; Lamb et al., 2013). No less important than this ATG7-mediated autophagic assembly is the function and regulation of Atg7 in natural and stressed pathophysiological conditions. Thanks to the dedication and contribution of numerous laboratories over the years, recent exciting findings on ATG7 have caused a paradigm shift in the field of ATG7-mediated autophagic regulation. A brief historical overview of select prior landmark investigations has been summarized in the timeline of Fig. 2. Given that these studies reflect the nature of ATG7’s intrinsic double-edged sword in development and disease, it is likely that excessive or deficient Atg7-mediated autophagy is harmful. Following advances in therapeutic manipulation of autophagy (Kroemer, 2015), it will be important to determine the specific and safe methods for pharmacologic fine-tuning of ATG7 activity.

Another cardinal question concerns personal medicine for accurate and rapid diagnosis of ATG7-related disease. The complex role of ATG7 seems to be highly structured in a spatiotemporal fashion rather than *ad libitum* (Behrends et al., 2010). The diverse roles of Atg7 in different settings summarized by this review may be attributed to selective Atg7-mediated autophagy at distinct organelle, cell, tissue, organ, and organism levels. An accurate understanding of the specified and delicate roles of Atg7-autophagy requires the in-depth knowledge of both the contextual extracellular cues and the intracellular responses. Thus, it will be interesting to search for the exact niches responsible for how Atg7 activity is encoded. It is also plausible that certain intricate forms of crosstalk interactions between Atg7-mediated autophagy and other autophagy-dependent or -independent pathways are responsible for shaping the versatile functions of Atg7. Therefore, it might be essential to identify and characterize the key coordinators of Atg7-mediated autophagy and other regulatory networks. Despite these challenges to be faced, academic and industry’s research progress in Atg7 offers new avenues towards refined autophagic mechanism and Atg7-based clinical treatment.

## **ABBREVIATIONS**

2D LC: two-dimensional liquid chromatography
Aβ: amyloid beta
ATG: autophagy-related
Atg7^−/−^: Atg7-deficient mice
Atg7^flox/flox^: Atg7-floxed mice
CryAB^R120G^: αB-crystallin R120G mutation
EndMT: endothelial-to-mesenchymal transition
FOXO: forkhead box O
HCV: hepatitis C virus
HIV: human immunodeficiency virus
HUVECs: human umbilical vein endothelial cells
iTRAQ: isobaric tags for relative and absolute quantitation
LC3: light chain 3
MEF: mouse embryonic fibroblast
MS/MS: tandem mass spectrometry
mTOR: mammalian target of rapamycin
NIH: National Institutes of Health
NSCLC: non-small cell lung cancer
PINK1: PTEN-induced kinase 1
siRNA: small interference RNA
VWF: Von Willebrand factor
WT: wild-type
